# Molecular subgroups of medulloblastoma: an international meta-analysis of transcriptome, genetic aberrations, and clinical data of WNT, SHH, Group 3, and Group 4 medulloblastomas

**DOI:** 10.1007/s00401-012-0958-8

**Published:** 2012-02-23

**Authors:** Marcel Kool, Andrey Korshunov, Marc Remke, David T. W. Jones, Maria Schlanstein, Paul A. Northcott, Yoon-Jae Cho, Jan Koster, Antoinette Schouten-van Meeteren, Dannis van Vuurden, Steven C. Clifford, Torsten Pietsch, Andre O. von Bueren, Stefan Rutkowski, Martin McCabe, V. Peter Collins, Magnus L. Bäcklund, Christine Haberler, Franck Bourdeaut, Olivier Delattre, Francois Doz, David W. Ellison, Richard J. Gilbertson, Scott L. Pomeroy, Michael D. Taylor, Peter Lichter, Stefan M. Pfister

**Affiliations:** 1 Division of Pediatric Neurooncology, German Cancer Research Center (DKFZ), Im Neuenheimer Feld 280, 69120 Heidelberg, Germany; 2 Clinical Cooperation Unit Neuropathology, German Cancer Research Center (DKFZ), Heidelberg, Germany; 3 Department of Pediatric Hematology and Oncology, Heidelberg University Hospital, Heidelberg, Germany; 4 Arthur and Sonia Labatt Brain Tumour Research Centre, Program in Developmental and Stem Cell Biology, Hospital for Sick Children, University of Toronto, Toronto, Canada; 5 Stanford University School of Medicine, Department of Neurology and Neurological Sciences, Stanford, USA; 6 Department of Oncogenomis, Academic Medical Center, Amsterdam, The Netherlands; 7 Department of Pediatric Oncology, Academic Medical Center, Amsterdam, The Netherlands; 8 Department of Pediatric Oncology/Hematology, Neuro-Oncology Research Group, Cancer Center Amsterdam, VU University Medical Center, Amsterdam, The Netherlands; 9 Northern Institute for Cancer Research, Newcastle University, Newcastle upon Tyne, UK; 10 Department of Neuropathology, Bonn University, Bonn, Germany; 11 Department of Pediatric Hematology and Oncology, University Medical Center Hamburg-Eppendorf, Hamburg, Germany; 12 Manchester Academic Health Science Centre, Manchester, UK; 13 Department of Pathology, University of Cambridge, Cambridge, UK; 14 Department of Oncology-Pathology, Karolinska Institute, Karolinska University Hospital, Stockholm, Sweden; 15 Department of Neuropathology, Medical University, Vienna, Austria; 16 Laboratoire de génétique et biologie des cancers, Institut Curie, Paris, France; 17 Department of Pediatric Oncology, Institut Curie and University Paris Descartes, Sorbonne Paris, France; 18 Department of Pathology, St. Jude Children’s Research Hospital, Memphis, USA; 19 Department of Developmental Neurobiology, St. Jude Children’s Research Hospital, Memphis, USA; 20 Department of Neurology, Children’s Hospital Boston, Harvard Medical School, Boston, USA; 21 Division of Neurosurgery, Hospital for Sick Children, University of Toronto, Toronto, Canada; 22 Division of Molecular Genetics, German Cancer Research Center (DKFZ), Heidelberg, Germany

**Keywords:** Medulloblastoma, Pediatric brain tumor, Subgroups, Meta-analysis

## Abstract

Medulloblastoma is the most common malignant brain tumor in childhood. Molecular studies from several groups around the world demonstrated that medulloblastoma is not one disease but comprises a collection of distinct molecular subgroups. However, all these studies reported on different numbers of subgroups. The current consensus is that there are only four core subgroups, which should be termed WNT, SHH, Group 3 and Group 4. Based on this, we performed a meta-analysis of all molecular and clinical data of 550 medulloblastomas brought together from seven independent studies. All cases were analyzed by gene expression profiling and for most cases SNP or array-CGH data were available. Data are presented for all medulloblastomas together and for each subgroup separately. For validation purposes, we compared the results of this meta-analysis with another large medulloblastoma cohort (*n* = 402) for which subgroup information was obtained by immunohistochemistry. Results from both cohorts are highly similar and show how distinct the molecular subtypes are with respect to their transcriptome, DNA copy-number aberrations, demographics, and survival. Results from these analyses will form the basis for prospective multi-center studies and will have an impact on how the different subgroups of medulloblastoma will be treated in the future.

**Electronic supplementary material:**

The online version of this article (doi:10.1007/s00401-012-0958-8) contains supplementary material, which is available to authorized users.

## Introduction

The embryonal brain tumor medulloblastoma is the most common malignant brain tumor in childhood. However, in several studies using transcriptional profiling we and others have shown that medulloblastoma is not a single disease, but in fact comprises a collection of clinically and molecularly diverse tumor subgroups [1, 9, 15, 19, 20, 25]. Two of these subgroups, characterized by either activated WNT or SHH signaling, consistently showed the most distinct genetic profiles. Recently, it was found that they also have different cellular origins [7]. Non-WNT/non-SHH tumors are more closely related to each other and the previously mentioned profiling studies reported on different numbers of subgroups within this group of medulloblastomas. Initially, three more subgroups were identified [9], characterized by elevated expression of neuronal differentiation genes (subgroups C and D), or the expression of photoreceptor genes (subgroups D and E). Another more recent study even identified four subgroups within the Non-WNT/Non-SHH group with subgroups c2 and c4 corresponding to the previously identified subgroups C and D [9], respectively [1]. For subgroup E according to Kool et al. [9], two subsets were identified (c1 and c5), which differed in gene expression patterns caused by the high frequency of *MYC* amplifications in c1 tumors. However, the current consensus in the medulloblastoma field is that there are only four core molecular subgroups in medulloblastoma, as recently agreed upon at a consensus meeting in Boston (see also the manuscript by Taylor et al. [24] in this issue). These four subgroups, which we will now call WNT, SHH, Group 3 and Group 4, are the same four subgroups as proposed in the study by Northcott et al. [15] and Remke et al. [19, 20]. It is important to note, however, that as larger cohorts will be analysed in the future, additional subtypes with specific genetic aberrations or other molecular or clinical properties might still be identified within each of these core subgroups, as was recently demonstrated for SHH medulloblastomas [14]. Having reached the consensus about the four major subgroups we have now re-analysed all the existing expression profiles from seven different studies ([1, 5, 9, 15, 19, 25]; McCabe et al., unpublished) and performed a meta-analysis of all available molecular and clinical data. Data for all medulloblastomas together and for each of the four subgroups separately are presented in this paper and compared with the data from another large cohort of medulloblastomas in which subgroup affiliation was determined by immunohistochemistry.

## Patients and methods

### Patients

Original data from seven studies with a total of 550 medulloblastoma patients were used for this study (Table S1). Information on gender, age at diagnosis, and histology was available for 523 patients (95%). Pathology was reviewed according to the 2007 WHO classification for central nervous system tumors [13]. One of these histological subtypes is characterized by extensive nodularity (MBEN). Although we acknowledge that these cases are different with respect to genetics, clinics and histology, there was only a single case in all seven studies classified as MBEN [15], which we have pooled with desmoplastic medulloblastoma in our study. We categorized the patients in three age groups: infants (aged <4 years), children (aged 4–16 years), and adults (aged >16 years). Information for metastatic stage (≥M1) at diagnosis was available for 432 patients (79%). Survival data were available for 388 patients (71%). The median follow-up time of survivors was 5.4 years (range 0.1–20.3 years). Data on whether patients received chemotherapy and/or radiotherapy were available for 234 patients (43%). For validation, we used the data from a largely independent medulloblastoma tissue micro array (TMA) cohort with tumors from 402 patients. Thirty-eight cases were also included in the Remke expression profiling series [19, 20]. All these patient samples were serially collected at the NN Burdenko Neurosurgical Institute (Moscow, Russia) between 1995 and 2007. Subgroup information for all tumors on the TMA was obtained by immunohistochemistry using antibodies for the subgroup-specific protein markers β-catenin (WNT), DKK1 (WNT), SFRP1 (SHH), NPR3 (Group 3), and KCNA1 (Group 4) as reported in [15, 19]. Information on gender, age at diagnosis, histology, metastatic stage at diagnosis and survival was available for all 402 patients (Table S1). As for the transcription profiling cohort pathology was reviewed according to the 2007 WHO criteria [13]. The median follow-up time of survivors in the TMA cohort was 3.6 years (range 0.3–17.0 years).

### Analyses

Medulloblastoma expression profiles generated on Affymetrix 133A [1, 25], Affymetrix 133plus 2.0 [5, 9]; McCabe et al., unpublished), Affymetrix exon 1.0 arrays [15], or Agilent arrays [19, 20], were available for all 550 patients. Data are accessible through the open access database R2 for visualization and analysis of microarray data (http://r2.amc.nl). Subgroup annotation for each dataset was obtained from semi non-negative matrix factorization (NMF) [6] using the 500 most differentially expressed genes. Array-CGH or SNP data were available for 383 medulloblastomas from five of the seven studies [1, 5, 9, 15, 19]. Overall survival was calculated from the date of diagnosis until death or last follow-up date. Univariate survival analysis was performed using the Kaplan–Meier method and log-rank test (SPSS 15.0). A multivariate Cox proportional hazards regression model, with overall survival as the dependent variable, was used to test the independency of each prognostic factor that was significant by univariate analysis. Two-sided *p* < 0.05 using 95% confidence interval was considered statistically significant.

## Results

### Patient characteristics in the gene expression profiling studies

The seven datasets used in this study were comparable regarding most patient characteristics (Table S1). Only the Thompson and Fattet series mainly included infants and children, whereas all other series also included adult medulloblastoma patients. The Remke series was even enriched for adults accounting for almost 50% in this cohort. In the McCabe series, only classic medulloblastomas were included. Of all patients, 21% were infants (age <4), 67% children (age 4–16) and 12% adults (age >16). In the Thompson series, an equal number of males and females were included, but all other series contained more males than females. One of the aims of the meta-analyses is also to overcome these cohort-specific biases. The median age of all patients was 7.3 years (range 0.3–52 years) (Table S1).

### Four molecular subtypes in medulloblastoma

**Fig. 1:**
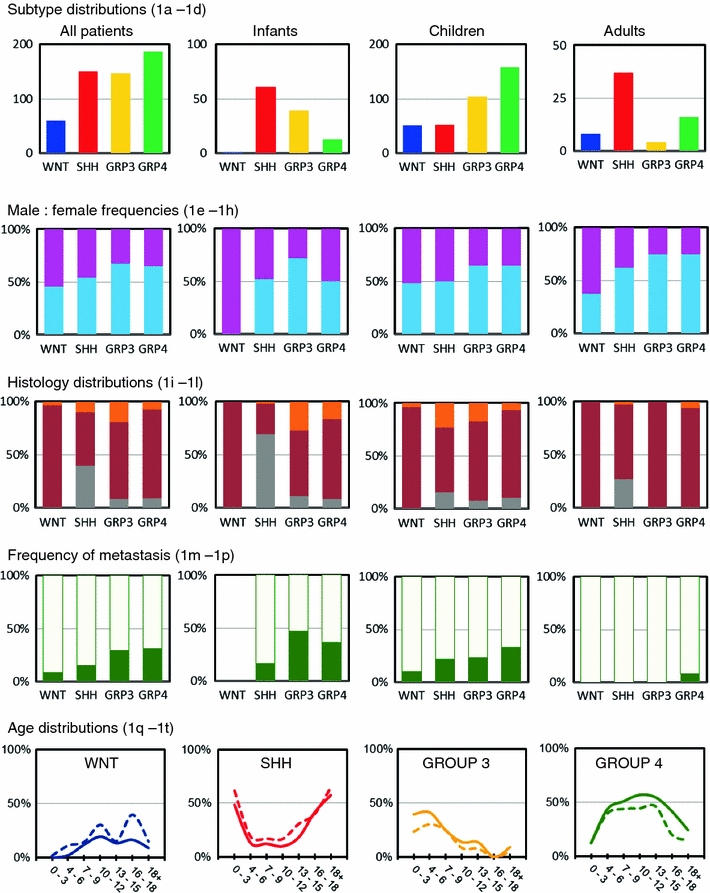
Demographic distribution of medulloblastoma subgroups. Subgroup distribution is shown for all medulloblastoma patients (**a**), infants (age <4 years) (**b**), children (age 4–16) (**c**), and adults (age >16) (**d**). *Numbers* on the *Y* axis indicate number of patients. Male:female frequencies are shown for all four subgroups in all patients (**e**), infants (**f**), children (**g**), and adults (**h**). Males are indicated in *blue*, females in *pink*. Distribution of histological subtype is shown for all four subgroups in all patients (**i**), infants (**j**), children (**k**), and adults (**l**). Classic histology is indicated in *dark red*, desmoplastic/extensive nodular histology in *gray*, and large cell/anaplastic histology in *orange*. Frequencies of metastasized (*green*) and non-metastasized (*light green*) cases are shown for all four subgroups in all patients (**m**), infants (**n**), children (**o**), and adults (**p**). Age distribution shown for males (*solid lines*) and females (*dotted lines*) is plotted for each of the four subgroups: WNT (**q**), SHH (**r**), Group 3 (**s**), and Group 4 (**t**). *Numbers* on the *Y* axis indicate the frequency of that particular subgroup within the indicated age group (in years) on the *X* axis among all patients

Group 4 tumors formed the largest group (34%) in this meta-analysis, followed by SHH (28%) and Group 3 tumors (27%). WNT tumors represented the smallest group (11%) (Fig. 1a). Distribution of these molecular subtypes was, however, significantly different between the three age groups (*p* < 0.001). Both among infants and adults, SHH tumors were most prominent and represented more than half of the cases, but in children they were much less frequent (14%) (Fig. 1b–d). WNT tumors were almost absent in infants (1%) and the frequency of Group 4 tumors was also much lower in this age group (11%). In contrast, Group 3 tumors were hardly found in adults (6%).

### Gender distribution

Overall, medulloblastoma affects males (M) about 1.5 times more often than females (F) [13], which was also evident from these combined series (Table S1). However, the M:F ratios were significantly different between the molecular subgroups (*p* = 0.008) (Fig. 1e–h). WNT and SHH tumors occurred almost equally in males and females, whereas Group 3 and Group 4 tumors clearly affected males about twice as often as females. This was found in all three age categories.

### Histology distribution

Most medulloblastomas were of classic histology (70%), followed by desmoplastic (16%) and large cell/anaplastic (LCA) histology (10%), but their frequencies varied between the different age categories (Fig. 1i–l). For instance, in infants the frequency of desmoplastic tumors was much higher (42%), but in children it was lower (9%). In adults, a very low frequency of LCA tumors was found (3%). Furthermore, there was a highly significant difference between the molecular subgroups and their occurrence within each histological variant (*p* < 0.001). Moreover, for each subgroup separately, the histological frequencies were also different between the three age categories. For instance, there is a strong association between desmoplastic histology and SHH tumors. In infants 39/44 (89%) and in adults all (*n* = 10) desmoplastic cases were classified as belonging to the SHH subgroup. In children, however, only 8/32 (25%) of the desmoplastic cases were classified as a SHH tumor. Nearly all (97%) WNT tumors had classic histology. Only 2/58 had LCA histology. Other LCA tumors were almost equally distributed over the other three molecular subgroups, but in infants they were almost all (10/13) classified as Group 3 tumors.

### Metastasis

Metastatic disease (M1–M4) at diagnosis was found in 103 of the 432 (24%) patients for whom the metastatic stage was known (Table S1). As expected according to previous reports [11, 20], in adults this percentage was much lower (2%), while there was not much difference between infants and children when the overall frequency of metastasis was considered between these age groups (30 and 26%, respectively). Among all patients, the highest frequency of metastatic disease at diagnosis was found for Group 3 (30%) and Group 4 tumors (31%) and these percentages were even higher in the infant group (47 and 36%, respectively) (Fig. 1m–p). For SHH tumors, metastatic disease was primarily found in infants (17%) and children (22%), but not in adults. For WNT tumors, metastasis was detected in 9% of cases and only in children.

### Age distribution

Almost half (44%) of all medulloblastomas were diagnosed in children between the age of 4 and 9 years, while 23% occurred in older children (10–16), 21% in infants (0–3), and 12% in adults (>16) (Table S1). The age distribution for each of the molecular subgroups differed dramatically (Fig. 1q–t). For instance, SHH tumors were most frequent in infants and adults and also Group 3 tumors were commonly found in infants, but not Group 4 or WNT tumors. These subgroups had their peak incidence later in childhood, at 5–13 or 10–12 years of age, respectively. No differences in age distribution were found between males and females.

### Cytogenetics

**Fig. 2:**
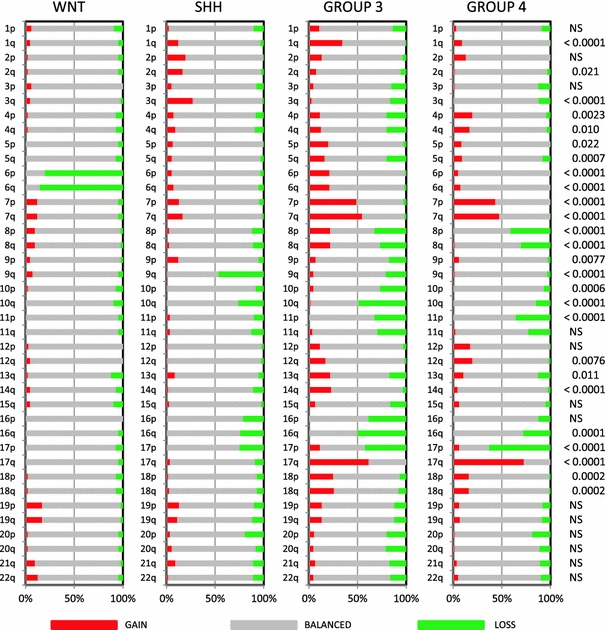
Overview of chromosomal aberrations in the four medulloblastoma subgroups. Array-CGH and SNP data were scored for loss (*green*), gain (*red*), or no change (*gray*) for all chromosomal arms. Results were plotted as frequencies at which these aberrations occurred within each molecular subgroup. *P* values on the right indicate whether there was a significant difference in the distribution of these frequencies across the four subgroups (Chi-square test). *NS* not significant

Analysis of the a-CGH and SNP profiling data, available for most cases in five of the seven medulloblastoma series, showed clear differences in chromosomal aberrations as has been reported for each of these series separately [1, 5, 9, 15, 19]. Complete or partial loss of chromosome 6 was found in 35/41 (85%) of WNT-driven tumors, but was nearly absent in all other subgroups (Fig. 2). Loss of 9q was most frequently detected in SHH tumors (47%), but was also found in Group 3 tumors (21%). Loss of 17p with or without concomitant 17q gain was most frequently found in Group 3 (loss 17p: 42%, gain 17q: 62%) and Group 4 (loss 17p: 63%, gain 17q: 73%). Loss of 17p only was also present in the SHH group (25%). Other chromosomal aberrations enriched in specific subgroups included 1q gain (Group 3, 35%), 3q gain (SHH, 27%), 7 gain (Group 3 and 4, 55 and 47%, respectively), 8(p) loss (Group 3 and 4, 33 and 41%, respectively), 8q gain (Group 3, 22%), 10q loss (most frequent in Group 3 (49%) but also present in SHH (26%) and Group 4 (15%)), 12(q) gain in Group 3 (17%) and Group 4 (20%), 16q loss (most frequent in Group 3, 50%), and 18 gain in Group 3 (26%) and Group 4 (16%).

### Survival analyses

**Fig. 3:**
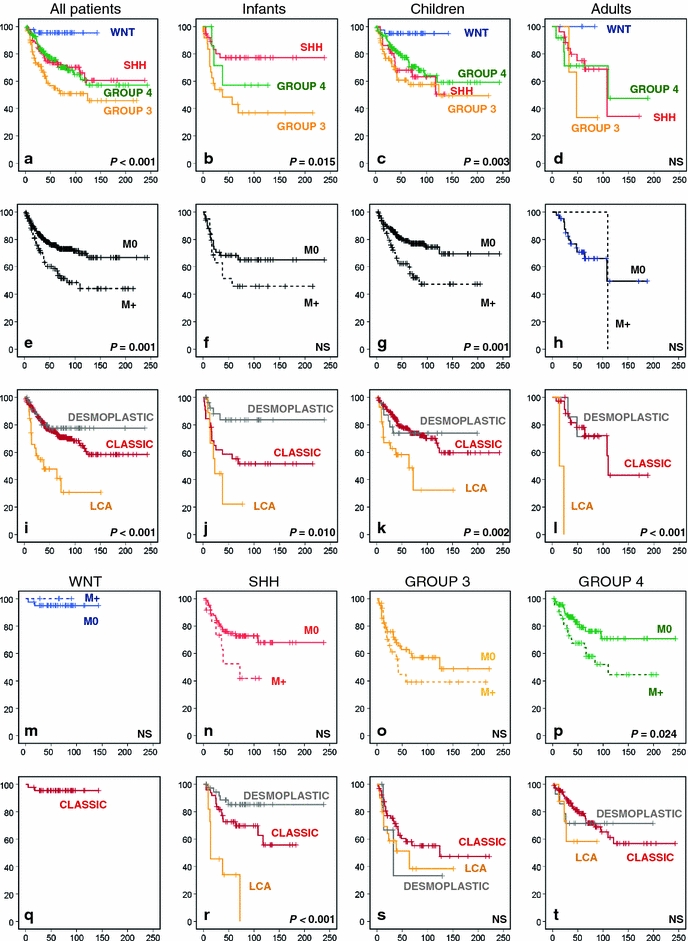
Overall survival (OS) analyses of molecular, clinical, and histological subgroups within the gene expression profiling cohort using Kaplan–Meier plots and log-rank tests. OS analysis of molecular subgroups among all patients (**a**), infants (**b**), children (**c**), and adults (**d**). OS analysis of metastasized (M1–M4, indicated as M+) versus non-metastasized (M0) cases, plotted for all patients (**e**), infants (**f**), children (**g**), and adults (**h**). OS analyses of classic, desmoplastic and LCA histological subgroups among all patients (**i**), infants (**j**), children (**k**), and adults (**l**). OS analysis of metastasized (M1–M4, indicated as M+) versus non-metastasized (M0) cases, plotted for each molecular subgroup: WNT (**m**), SHH (**n**), Group 3 (**o**), and Group 4 (**p**). OS analyses of classic, desmoplastic and LCA histological subgroups plotted for each molecular subgroup: WNT (**q**), SHH (**r**), Group 3 (**s**), and Group 4 (**t**). *Numbers* on the *Y* axis indicate the fraction of surviving patients. *Numbers* on the *X* axis indicate the follow-up time in months. *NS* not significant

Survival analyses for the combined series for which survival data were available showed a clear and significant difference in overall survival (OS) between the four molecular subgroups. This was not only the case for all patients together but also when infants or children were analysed separately. Only for the adult category the differences did not reach significance (Fig. 3a–d). Both in children and in adults, WNT tumors had by far the best outcome with a 5- and 10-year OS of 95% in children (*n* = 39) and a 5-year OS of 100% in adults (*n* = 5). The worst outcome in all age categories was seen for patients with Group 3 tumors (Infants 5- and 10-year OS 45 and 39%, respectively; children 5- and 10-year OS 58 and 50%, respectively). In adults, 2/3 patients having a Group 3 medulloblastoma died. While the outcome for patients with both Group 3 and 4 tumors was more or less similar in all age categories, SHH tumors clearly had a better outcome in infants (5- and 10-year OS 77%) compared to children (5- and 10-year OS 68 and 51%, respectively) and adults (5- and 10-year OS 75 and 34%, respectively). This is likely associated with the high frequency of desmoplastic histology among SHH tumors in infants, as it has been demonstrated that desmoplastic/extensive nodular histology in this age group is a marker of favorable prognosis [21]. Histological subtyping indeed showed that especially desmoplastic histology in infants predicts a very good outcome (5- and 10-year OS 84%). In contrast, large cell anaplastic (LCA) histology predicts a very poor outcome in all age categories (infants 5-year OS 22%; children 10-year OS 32%; in adults both patients with LCA histology died) (Fig. 3i–l). Histological subtyping is also important within molecular subgroups, especially for SHH tumors where the histological subtypes show a large difference in outcome (Fig. 3q–t). Infants and children with metastatic dissemination at diagnosis in general have a worse outcome than patients without, but only in children this difference was significant (Fig. 3e–h). Also within SHH, Group 3 or Group 4 tumors we see that patients with metastatic dissemination do worse than those without, but only for Group 4 patients this difference in overall survival was significant (Fig. 3n–p). Interestingly, all four patients with a WNT tumor and metastatic disease survived (Fig. 3m).

### MYC family oncogene amplifications

**Fig. 4:**
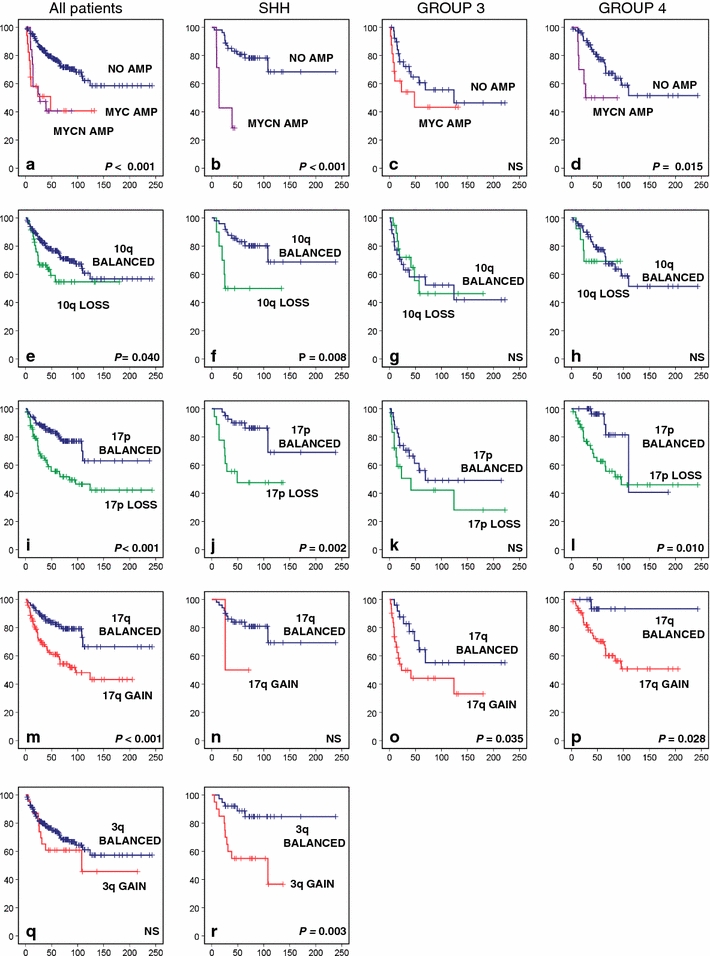
Overall survival (OS) analyses of cytogenetic subgroups using Kaplan–Meier plots and log-rank tests for statistical significance. Data are shown for all patients within the gene expression profiling cohort, for the SHH subgroup, for Group 3, and for Group 4. OS analysis of patients having a MYC or MYCN amplification versus patients not having these amplifications. **e**–**h** OS analysis of patients harboring 10q loss versus patients with a balanced 10q. **i**–**l** OS analysis of patients harboring 17p loss versus patients with a balanced 17p. **m**–**p** OS analysis of patients harboring 17q gain versus patients with a balanced 17q. **q**–**r** OS analysis of patients harboring 3q gain versus patients with a balanced 3q. Frequency of 3q gain within Group 3 and Group 4 was too low to perform survival analyses. *Numbers* on the *Y* axis indicate the fraction of surviving patients. *Numbers* on the *X* axis indicate the follow-up time in months. *NS* not significant

High-level gene amplifications are overall rarely observed in medulloblastoma, but when they do occur, they most frequently affect the *MYC* or *MYCN* oncogenes and only in a very few cases the related *MYCL* gene. Almost all *MYC* amplifications were identified in Group 3 tumors (24/27). The other three cases were found in SHH (*n* = 1) or Group 4 tumors (*n* = 2). In contrast, *MYCN* amplifications mostly occurred in either SHH (*n* = 10) or Group 4 tumors (*n* = 12) and only rarely in Group 3 tumors (*n* = 3). All three *MYCL* amplifications occurred in SHH tumors. Patients having either a *MYC* or *MYCN* amplification in their tumor clearly have a significantly worse outcome compared to cases without amplification (Fig. 4a). This is true for all patients and also in a subgroup-specific manner, except for Group 3 tumors. In this subgroup, patients without *MYC* amplification in their tumor do equally poor as those carrying this alteration (Fig. 4a–d).

### Survival analyses of cytogenetic groups

Other cytogenetic aberrations that were found to be significantly associated with outcome either in all patients or within particular molecular subgroups included gain of 3q, loss of 10q, loss of 17p, and gain of 17q (Fig. 4e–r). Gain of 3q and loss of 10q was associated with poor outcome only in SHH tumors. Chromosome 17 aberrations, most frequently found in Group 3 and 4 tumors, were also associated with a significantly worse outcome within these subgroups, and most clearly for Group 4 tumors. Loss of 17p, although much less frequent in SHH tumors, was also associated with an unfavorable outcome in this subgroup (Fig. 4j).

### Multivariate analyses

Multivariate COX analyses were performed for the entire cohort and for each subgroup separately using only the factors that were significant by univariate survival analysis (Table S2). For all patients, molecular subgrouping was shown to be an independent prognostic factor with relative risks of the various subgroups ranging from 1.9 to 4.1 compared to the WNT group (Table 1). *MYC(N)* amplifications and loss of 17p also remained as independent prognostic factors in the multivariate analysis for all groups. For SHH tumors, both LCA histology and gain of 3q were independent prognostic factors of adverse outcome, while for Group 3 and 4 tumors, only chromosome 17 aberrations (gain of 17q or loss of 17p, respectively) remained significant (Table 1). As different treatment protocols may have influenced these survival analyses, we also performed multivariate COX analyses including only patients for we knew that they had received radio- and chemotherapy. Furthermore, as data on treatment were limited for some series, we also performed the same analyses for non-infants only, assuming that most of them received radio- and chemotherapy. Results of these analyses, presented in Tables S2 and S3, show that even after correcting for treatment or age factors like histology, *MYCN* amplification and gain of 3q remained significant for all patients, and in the SHH subgroup. Chromosome 17 aberrations remained significant after these corrections for the entire cohort, SHH medulloblastomas and Group 4.

**Table 1:** Multivariate overall survival analyses

Prognostic factor	RR	CI low	CI high	*P value*
All patients (*n* = 204)				
Subgroup				0.036
SHH vs. WNT	1.9	0.4	8.9	0.4
Group 3 vs. WNT	4.1	0.9	18.2	0.065
Group 4 vs. WNT	1.9	0.4	8.7	0.4
MYC(N) amplification yes vs. no	3.4	1.7	6.5	<0.001
17p loss yes vs. no	2.4	1.4	4.3	0.002
SHH medulloblastomas (*n* = 54)				
Histology				0.001
Desmoplastic vs. Classic	0.3	0.1	1.1	0.071
LCA vs. Classic	8.9	2.0	40.6	0.005
3q gain yes vs. no	4.5	1.5	13.9	0.008
Group 3 medulloblastomas (*n* = 44)				
17q gain yes vs. no	2.6	1.0	6.6	0.049
Group 4 medulloblastomas (*n* = 79)				
17p loss yes vs. no	3.6	1.2	10.8	0.020

### Data validation using an independent medulloblastoma cohort

We used data from an independent large medulloblastoma tissue microarray (TMA) cohort (*n* = 402) to compare the results obtained from the GEP series. The TMA cohort was analyzed by immunohistochemistry only and subgroup annotation was obtained using specific-marker antibodies as described in [15, 19]. Data presented in Fig. S2 show that both the GEP and TMA cohorts were very similar regarding distribution of molecular and histological subgroups and the frequency of metastases at diagnosis within each molecular subgroup and within the different age categories. Only gender ratios for especially WNT and SHH tumors appeared to be slightly different for the TMA cohort, with more females in the WNT group and more males in the SHH group. Finally, we also performed overall survival analyses for this TMA cohort similar to the analyses for the GEP cohort shown in Fig. 4a–t. Data presented in Fig. S3 show that in general these survival analyses look very similar to the ones from the GEP cohort, but they revealed also some interesting differences. For instance, patients with SHH medulloblastomas in the TMA cohort showed a much better overall survival (5- and 10-year OS 87 and 77%, respectively), which was mainly due to a better survival of children (5- and 10-year OS 90 and 80%, respectively) and adults (5- and 10-year OS 85 and 74%, respectively) in this molecular subgroup. In contrast, patients with Group 3 medulloblastomas did much worse. No patient with a Group 3 tumor in this series survived longer than 124 months. Moreover, patients with WNT or Group 4 tumors, and especially those in adults, did worse than the ones in the GEP cohort.

## Discussion

The meta-analysis presented here represents the largest series of biology data on medulloblastoma reported so far. The data clearly demonstrate that medulloblastoma is not a single disease. The four major subgroups (WNT, SHH, Group 3, and Group 4) differ in many aspects. They are transcriptionally, genetically, demographically, clinically, and prognostically distinct, confirming earlier reports in smaller series [1, 3, 9, 15, 19, 25]. Most likely, they will also have different cellular origins, as has already been shown for the WNT and SHH subgroups [7, 8, 23, 26]. The cellular origin of Group 3 and 4 medulloblastomas is still unknown. Several of the earlier profiling studies showed that there might even be five or six subgroups of medulloblastoma [1, 9, 25], with further subdivisions of Group 3 and Group 4. An analysis performed on the combined GEP cohorts under the assumption that there were five or six subgroups showed that there are indeed subsets present within these subgroups with transcriptional and genetic differences, but demographically they were not different (data not shown). Collectively, these data demonstrate that there are only four core disease subgroups of medulloblastoma, with a yet unknown number of subsets within each subgroup. Subsets also exist within the SHH subgroup as we and others recently demonstrated [14, 18]. These subsets show transcriptional and genetic differences and seem to be associated with the different age groups (infants vs. adults; [14]) that exist within the SHH subgroup and with the presence of *P53* mutations [18]. Potentially, they could actually represent different disease variants with different cellular origins, which might explain the bimodal age distribution of SHH medulloblastomas. The meta-analysis data also show that prognostic factors like metastatic stage, histology, *MYC* and *MYCN* amplifications, 10q loss, 17p loss, and 17q gain, previously reported for medulloblastoma as a single disease [11, 12, 17], remain prognostic in these combined series of all patients. However, our data now show for the first time how they all perform in the context of different subgroups. For instance, the observation that medulloblastomas with chromosome 17 aberrations have an adverse outcome is due to the fact that they are most frequent in Group 3 and Group 4 medulloblastomas, which fare worse than the WNT and SHH subgroups. However, even within these subgroups, loss of 17p and/or gain of 17q remain independent prognostic factors for SHH, Group 3 and 4. Other factors, which are clearly prognostic for the entire medulloblastoma cohort, such as histology or metastasis, are barely prognostic in specific subgroups and most of them do not hold up in the multivariate analysis. Only for the SHH subgroup does histology remain an independent prognostic factor, and we have identified gain of 3q as a novel independent prognostic factor for this subgroup. *MYC* and *MYCN* amplifications also predict an unfavourable outcome in the entire cohort (Fig. 4a), in line with previous publications [3, 11, 17, 22]. However, *MYC* amplification, most frequent in Group 3 medulloblastomas, is not prognostic within this subgroup (Fig. 4c). In contrast, *MYCN* amplification, mostly occurring in SHH or Group 4 medulloblastomas, is still prognostic in both of these subgroups (Fig. 4b, d), but did not hold up in the multivariate analyses (Table 1). Only after correcting for age (excluding infants) *MYCN* amplification remains prognostic within the SHH subgroups (Table S3). Therefore, medulloblastoma subgrouping is by far the best factor in terms of prognostication identified to date, but there is now a need for identifying better prognostic markers within each of the subgroups. A good example of such a subgroup-specific biomarker is the recently identified FSTL5 protein [19]. Immunopositivity of FSTL5 identified a large group of patients at high risk across all medulloblastomas, but more importantly, also within Group 3 and 4 patients.

One drawback in the survival analyses performed in this meta-analysis is the fact that the patients contained in each of the different GEP cohorts come from different studies, and have been treated in multiple centers according to different protocols. This is also demonstrated by the overall survival of the four subgroups in the GEP cohort in comparison with that in the TMA cohort. All tumors in the TMA cohort come from patients treated in a single institute according to standardized therapeutic protocols of the German HIT study group. Interestingly, in this TMA cohort, patients with SHH medulloblastomas had a much better outcome compared to the SHH medulloblastomas in the combined GEP cohort, whereas especially patients with Group 3 medulloblastomas had a much worse prognosis. Furthermore, WNT medulloblastomas, reported in several studies as having a very good outcome [2–4, 15, 17], which is confirmed in the meta-analyses of the GEP cohorts, do not have such a good outcome in adults of the TMA cohort. One of the reasons explaining these differences in overall survival for the different subgroups between the GEP and TMA cohorts could be that in general medulloblastoma patients represented on the TMA cohort received less intensive therapies compared to most other patients present in the GEP cohorts. As illustrated in another paper in this issue [10], even *MYCN* amplified cases in the SHH subgroup have a better outcome when receiving less intensive therapies. These data suggest that most, if not all SHH medulloblastoma patients may benefit from a less intensive protocol, but other subgroups, and in particular Group 3 tumors, may not. Prospective studies targeting specific subgroups should aim to resolve this question. Future clinical trials will require reliable and reproducible methods to subgroup clinical medulloblastoma samples in a fast way. For this, the recently developed NanoString assay can be used, which predicts the tumor specific subgroup with high accuracy, based on the expression level of 22 subgroup-specific signature genes [16]. Alternatively, a panel of immunohistochemistry-based markers can be assessed, as demonstrated in previous publications [3, 15, 19]. As yet, the most reliable method to attribute patients to the four subgroups has still to be decided, but efforts are ongoing to address this question.

In summary, we consistently find four core molecular subgroups of medulloblastoma across all published datasets which are as distinct as different tumor entities and, therefore, should be regarded as such. Thus, future studies of medulloblastoma should accommodate this new clinically useful knowledge for optimizing trial design and treatment protocols.

## Electronic supplementary material

Below is the link to the electronic supplementary material.

Supplementary material 1 (DOCX 39 kb)

Supplementary material 2 (PPTX 746 kb)
