# Supplementary material for: Molecular subgroups of medulloblastoma: an international meta-analysis of transcriptome, genetic aberrations, and clinical data of WNT, SHH, Group 3, and Group 4 medulloblastomas
Source: Acta Neuropathol. 2012 Feb 23;123(4):473–84. doi: 10.1007/s00401-012-0958-8 (PMC3306778; doi:10.1007/s00401-012-0958-8)
Supplement: Supplementary file 1 — Supplementary material 1 (DOCX 39 kb) [file 401_2012_958_MOESM1_ESM.docx]

| **Table S1. Patient characteristics** | | |  |  |  |  |  |  |  |
| --- | --- | --- | --- | --- | --- | --- | --- | --- | --- |
|  |  |  |  |  |  |  |  |  |  |
| **Data set** | **Thompson** | **Kool** | **Fattet** | **Northcott** | **Cho** | **Remke** | **Mccabe** | **All GEP** | **Korshunov TMA** |
| Total no. cases | 46 | 61 | 56 | 102 | 187 | 64 | 34 | 550 | 402 |
| **Gender** |  |  |  |  |  |  |  |  |  |
| Male | 23 | 39 | 36 | 63 | 113 | 39 | 18 | 331 | 254 |
| Female | 23 | 22 | 20 | 39 | 74 | 25 | 16 | 219 | 148 |
| ND | 0 | 0 | 0 | 0 | 0 | 0 | 0 | 0 | 0 |
| **Age at diagnosis** |  |  |  |  |  |  |  |  |  |
| Average age | 7.1 | 8.6 | 7.4 | 9.5 | 7.8 | 17.5 | 11.9 | 9.5 | 13.6 |
| Median age | 6.5 | 6.0 | 7.7 | 7.0 | 6.6 | 13.0 | 9.2 | 7.3 | 10.0 |
| Age range | 1 - 16.7 | 1.5 - 35.3 | 0.3 - 18 | 0.9 - 39 | 0.9 - 43.9 | 1 - 52 | 0.6 - 41.0 | 0.3 - 52 | 0.6 - 52 |
| **Age groups** |  |  |  |  |  |  |  |  |  |
| Infants (< 4) | 14 | 17 | 11 | 19 | 41 | 5 | 6 | 113 | 54 |
| Children (4 -16) | 32 | 36 | 44 | 68 | 133 | 30 | 20 | 363 | 236 |
| Adults (> 16) | 0 | 6 | 1 | 14 | 7 | 29 | 8 | 65 | 112 |
| ND | 0 | 2 | 0 | 1 | 6 | 0 | 0 | 9 | 0 |
| **Histology** |  |  |  |  |  |  |  |  |  |
| Classic | 20 | 45 | 40 | 76 | 123 | 49 | 34 | 387 | 299 |
| Desmoplastic | 14 | 13 | 8 | 17 | 27 | 7 | 0 | 86 | 61 |
| LC/A | 12 | 1 | 3 | 8 | 24 | 8 | 0 | 56 | 42 |
| ND | 0 | 2 | 5 | 1 | 13 | 0 | 0 | 21 | 0 |
| **Staging** |  |  |  |  |  |  |  |  |  |
| M0 | 26 | 42 | 38 | 10 | 138 | 46 | 29 | 329 | 279 |
| M+ | 20 | 15 | 18 | 4 | 24 | 18 | 4 | 103 | 123 |
| M1 | 7 | 7 | 5 | 0 | 3 | 5 | 0 | 27 | 21 |
| ≥M2 | 13 | 8 | 13 | 0 | 16 | 13 | 4 | 67 | 102 |
| M+ (stage not specified)  ND | 0  0 | 0  4 | 0  0 | 4  88 | 5  25 | 0  0 | 0  1 | 9  118 | 0  0 |
| **Molecular subgroups** |  |  |  |  |  |  |  |  |  |
| WNT | 6 | 9 | 4 | 9 | 14 | 15 | 3 | 60 | 36 |
| SHH | 13 | 14 | 12 | 31 | 52 | 20 | 11 | 153 | 153 |
| Group 3 | 14 | 16 | 15 | 31 | 55 | 9 | 9 | 149 | 71 |
| Group 4 | 13 | 22 | 25 | 31 | 66 | 20 | 11 | 188 | 142 |

**Table S2** Univariate survival analyses of the GEP cohort analyzed for all patients and for the SHH, Group 3 and Group 4 subgroups, including all patients, only non-infants, or only those patients who received radiotherapy (RTX) and chemotherapy (CTX).

**All patients**

|  | **all** | | **non-infants** | | **all who received RTX + CTX** | |
| --- | --- | --- | --- | --- | --- | --- |
| ***Prognostic factor*** | ***N*** | ***P value*** | ***N*** | ***P value*** | ***N*** | ***P value*** |
| Subgroup | 376 | < 0.001 | 310 | 0.001 | 176 | 0.016 |
| Histology | 369 | < 0.001 | 303 | < 0.001 | 174 | < 0.001 |
| Metastasis | 366 | 0.001 | 298 | 0.001 | 175 | 0.030 |
| MYC(N) amplification | 245 | < 0.001 | 204 | < 0.001 | 146 | < 0.001 |
| 3q gain | 222 | NS | 181 | 0.014 | 130 | NS |
| 10q loss | 231 | 0.040 | 190 | NS | 137 | NS |
| 17p loss | 224 | 0.000 | 184 | < 0.001 | 131 | < 0.001 |
| 17q gain | 225 | 0.000 | 185 | 0.002 | 132 | < 0.001 |

**SHH**

|  | **all** | | **non-infants** | | **all who received RTX + CTX** | |
| --- | --- | --- | --- | --- | --- | --- |
| ***Prognostic factor*** | ***N*** | ***P value*** | ***N*** | ***P value*** | ***N*** | ***P value*** |
|  |  |  |  |  |  |  |
| Histology | 99 | < 0.001 | 61 | < 0.001 | 44 | < 0.001 |
| Metastasis | 95 | NS | 57 | NS | 43 | 0.010 |
| MYCN amplification | 61 | < 0.001 | 41 | < 0.001 | 34 | 0.001 |
| 3q gain | 58 | 0.003 | 38 | 0.001 | 32 | NS |
| 10q loss | 59 | 0.008 | 39 | NS | 33 | NS |
| 17p loss | 59 | 0.002 | 39 | 0.001 | 33 | 0.002 |
| 17q gain | 53 | NS | 34 | NS | 28 | NS |

**Group 3**

|  | **All** | | **non-infants** | | **all who received RTX + CTX** | |
| --- | --- | --- | --- | --- | --- | --- |
| ***Prognostic factor*** | ***N*** | ***P value*** | ***N*** | ***P value*** | ***N*** | ***P value*** |
|  |  |  |  |  |  |  |
| Histology | 91 | NS | 71 | NS | 43 | NS |
| Metastasis | 93 | NS | 72 | NS | 43 | NS |
| MYC amplification | 56 | NS | 44 | NS | 34 | NS |
| 3q gain | 52 | ND | 37 | ND | 30 | ND |
| 10q loss | 55 | NS | 41 | NS | 33 | NS |
| 17p loss | 54 | NS | 40 | NS | 32 | NS |
| 17q gain | 56 | 0.035 | 42 | NS | 34 | NS |

**Group 4**

|  | **all** | | **non-infants** | | **all who received RTX + CTX** | |
| --- | --- | --- | --- | --- | --- | --- |
| ***Prognostic factor*** | ***N*** | ***P value*** | ***N*** | ***P value*** | ***N*** | ***P value*** |
|  |  |  |  |  |  |  |
| Histology | 133 | NS | 126 | NS | 80 | NS |
| Metastasis | 132 | 0.024 | 125 | 0.042 | 82 | NS |
| MYCN amplification | 89 | 0.015 | 84 | 0.011 | 67 | NS |
| 3q gain | 82 | ND | 77 | ND | 61 | ND |
| 10q loss | 86 | NS | 81 | NS | 64 | NS |
| 17p loss | 81 | 0.010 | 76 | 0.004 | 59 | 0.048 |
| 17q gain | 85 | 0.028 | 80 | 0.011 | 63 | NS |

**Table S3** Multivariate overall survival analyses including only non-infants or only patients who received radiotherapy (RTX) and chemotherapy (CTX).

| **All non-infants** |  |  |  |  |
| --- | --- | --- | --- | --- |
|  |  |  |  |  |
| ***Prognostic factor*** | ***RR*** | ***CI low*** | ***CI high*** | ***P value*** |
|  |  |  |  |  |
| **All non-infants (n= 159)** |  |  |  |  |
| MYC(N) amplification *yes vs no* | 4.3 | 1.9 | 9.6 | < 0.001 |
| 3q gain *yes vs no* | 6.1 | 2.4 | 15.0 | < 0.001 |
| 17q gain *yes vs no* | 3.8 | 1.8 | 8.0 | < 0.001 |
|  |  |  |  |  |
| **SHH medulloblastomas (n = 31)** | |  |  |  |
| MYCN amplification *yes vs no* | 4.3 | 1.9 | 9.6 | 0.040 |
| 3q gain *yes vs no* | 6.1 | 2.4 | 15.0 | 0.045 |
| 17p loss *yes vs no* | 3.8 | 1.8 | 8.0 | 0.028 |
|  |  |  |  |  |
| **Group 4 medulloblastomas (n = 74)** | |  |  |  |
| 17p loss *yes vs no* | 4.8 | 1.4 | 16.8 | 0.012 |
|  |  |  |  |  |
|  |  |  |  |  |
|  |  |  |  |  |

| **All patients who received RTX and CTX** | | |  |  |
| --- | --- | --- | --- | --- |
|  |  |  |  |  |
| ***Prognostic factor*** | ***RR*** | ***CI low*** | ***CI high*** | ***P value*** |
|  |  |  |  |  |
| **All patients (n= 128)** |  |  |  |  |
| Histology |  |  |  | 0.030 |
| LCA vs classic | 3.8 | 1.4 | 10.1 | 0.008 |
| 17q gain *yes vs no* | 3.5 | 1.6 | 7.8 | 0.002 |
|  |  |  |  |  |
| **SHH medulloblastomas (n = 31)** | |  |  |  |
| Histology |  |  |  | 0.036 |
| Desmoplastic vs Classic | 0.2 | 0.0 | 1.8 | 0.150 |
| LCA vs Classic | 17.1 | 1.1 | 273.8 | 0.045 |
| 17p loss *yes vs no* | 21.3 | 2.0 | 223.0 | 0.011 |
|  |  |  |  |  |
| **Group 4 medulloblastomas (n = 58)** | |  |  |  |
| 17p loss *yes vs no* | 3.3 | 1.0 | 11.4 | 0.060 |
|  |  |  |  |  |

**Legends Supplementary Figures**

**Figure S1.** Non-negative matrix factorization (NMF) analysis for all gene expression profilingseries used in the meta-analysis using the 500 most differentially expressed genes (by standard deviation) within each data set. Subgroup annotations are indicated at the bottom.

**Figure S2.** Demographic distribution of medulloblastoma subgroups is compared between gene expression profiling (GEP) and tissue microarray (TMA) cohorts. Data are also shown for both cohorts combined and for all patients, for infants (aged < 4 yrs), for children (4 - 16), and for adults (>16). **a.** Subgroup distribution shown as fractions in pie charts. **b.** Subgroup distribution shown in absolute numbers. Numbers on the Y-axis indicate number of patients. **c.** Male : female ratios. Males are indicated in blue, females in pink. **d.** Distribution of histopathological subytpes. Classic histology is indicated in dark red, desmoplastic/extensive nodular histology in grey, and large cell/anaplastic histology in orange. **e.** Frequencies of metastasized (green) and non-metastasized (light green) cases are shown for all four subgroups.

**Figure S3.** Overall survival (OS) analyses of molecular, clinical, and histological subgroups within the tissue microarray cohort using Kaplan Meier plots and log rank. **a – d.** OS analysis of molecular subgroups among all patients (a), infants (b), children (c), and adults (d). **e – h.** OS analysis of metastatic positive (M1 – M4, indicated as M+) versus metastatic negative (M0) cases, plotted for all patients (e), infants (f), children (g), and adults (h). **i – l.** OS analyses of classic, desmoplastic and LCA histological subgroups among all patients (i), infants (j), children (k), and adults (l). **m – p.** OS analysis of metastasized (M1 – M4, indicated as M+) versus non-metastasized (M0) cases, plotted for each molecular subgroup: WNT (m), SHH (n), Group 3 (o), and Group 4 (p). **q – t.** OS analyses of classic, desmoplastic and LCA histological subgroups plotted for each molecular subgroup: WNT (q), SHH (r), Group 3 (s), and Group 4 (t). Numbers on the Y-axis indicate the fraction of surviving patients. Numbers on the X-axis indicate the follow-up time in months. NS = not significant.
