# Supplementary material for: Molecular subgroups of medulloblastoma: an international meta-analysis of transcriptome, genetic aberrations, and clinical data of WNT, SHH, Group 3, and Group 4 medulloblastomas
Source: Acta Neuropathol. 2012 Feb 23;123(4):473–84. doi: 10.1007/s00401-012-0958-8 (PMC3306778; doi:10.1007/s00401-012-0958-8)
Supplement: Supplementary file 2 — Supplementary material 2 (PPTX 746 kb) [file 401_2012_958_MOESM2_ESM.pptx]

## Slide 1
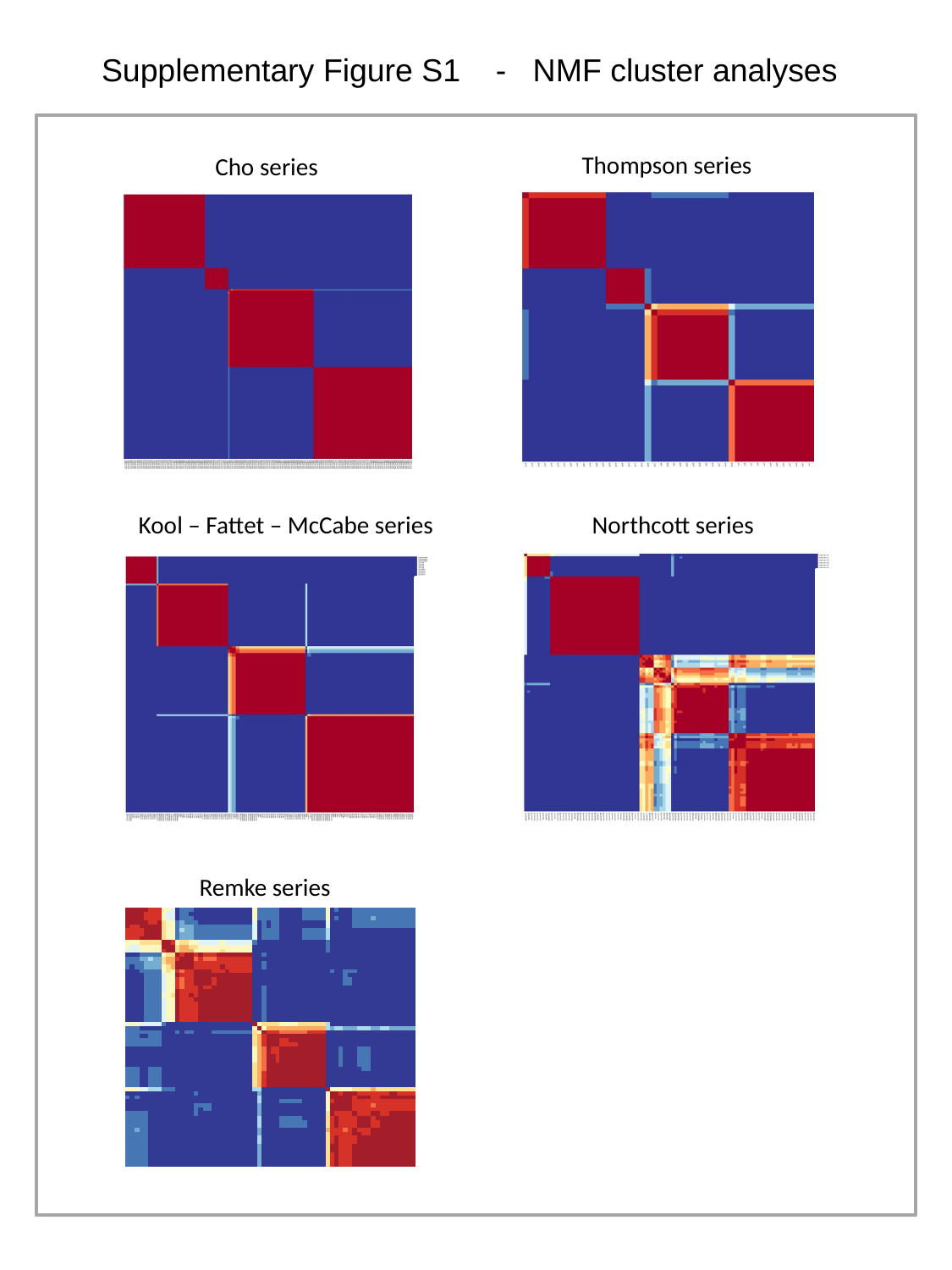

Supplementary Figure S1 - NMF cluster analyses
Thompson series
Cho series
Kool – Fattet – McCabe series
Northcott series
Remke series

## Slide 2
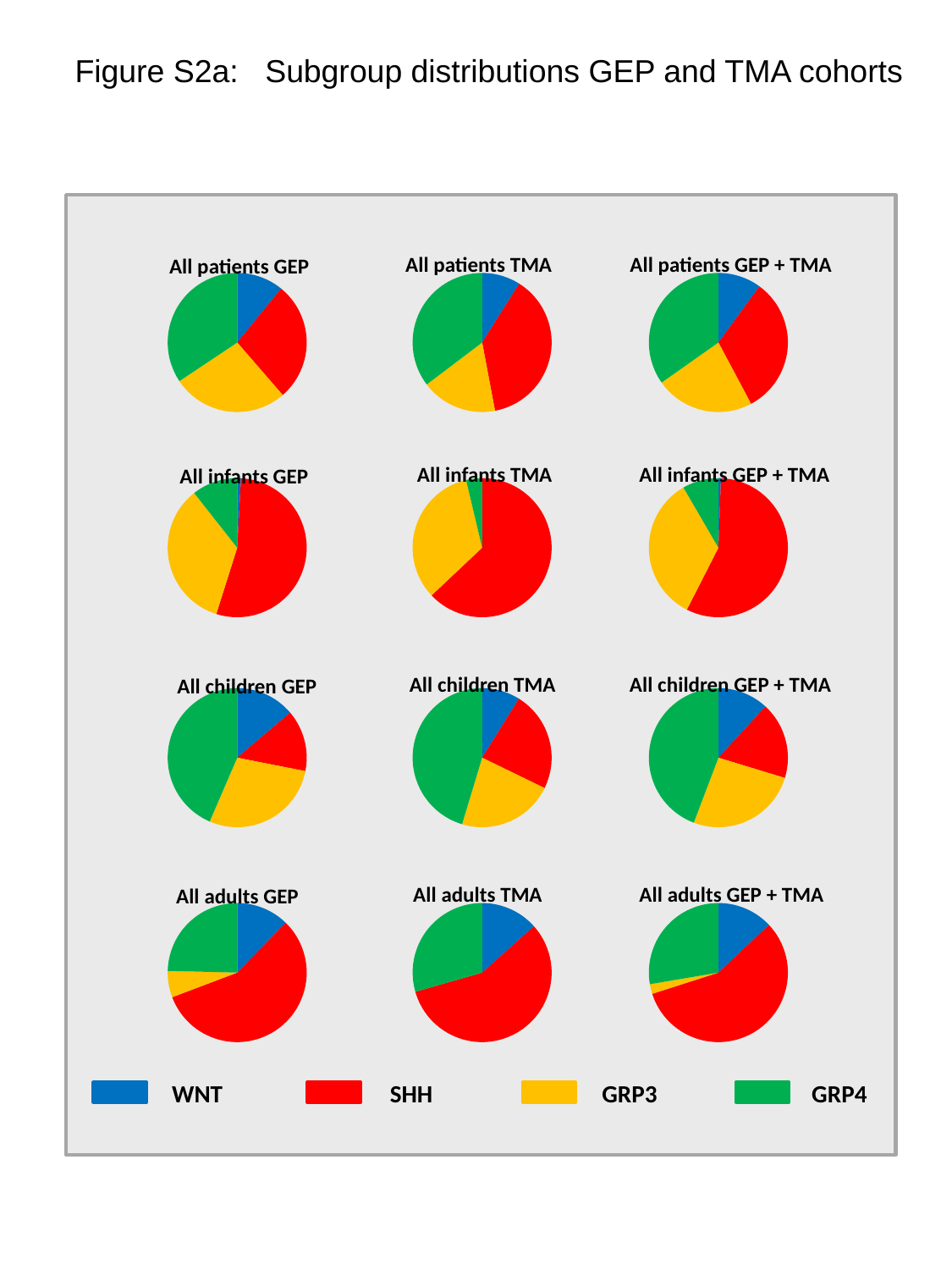

Figure S2a: Subgroup distributions GEP and TMA cohorts
All patients TMA
All patients GEP + TMA
All patients GEP
### Chart
| Category | |
|---|---|
### Chart
| Category | |
|---|---|
### Chart
| Category | |
|---|---|All infants TMA
All infants GEP + TMA
All infants GEP
### Chart
| Category | |
|---|---|
### Chart
| Category | |
|---|---|
### Chart
| Category | |
|---|---|All children TMA
All children GEP + TMA
All children GEP
### Chart
| Category | |
|---|---|
### Chart
| Category | |
|---|---|
### Chart
| Category | |
|---|---|All adults TMA
All adults GEP + TMA
All adults GEP
### Chart
| Category | |
|---|---|
### Chart
| Category | |
|---|---|
### Chart
| Category | |
|---|---|WNT
SHH
GRP3
GRP4

## Slide 3
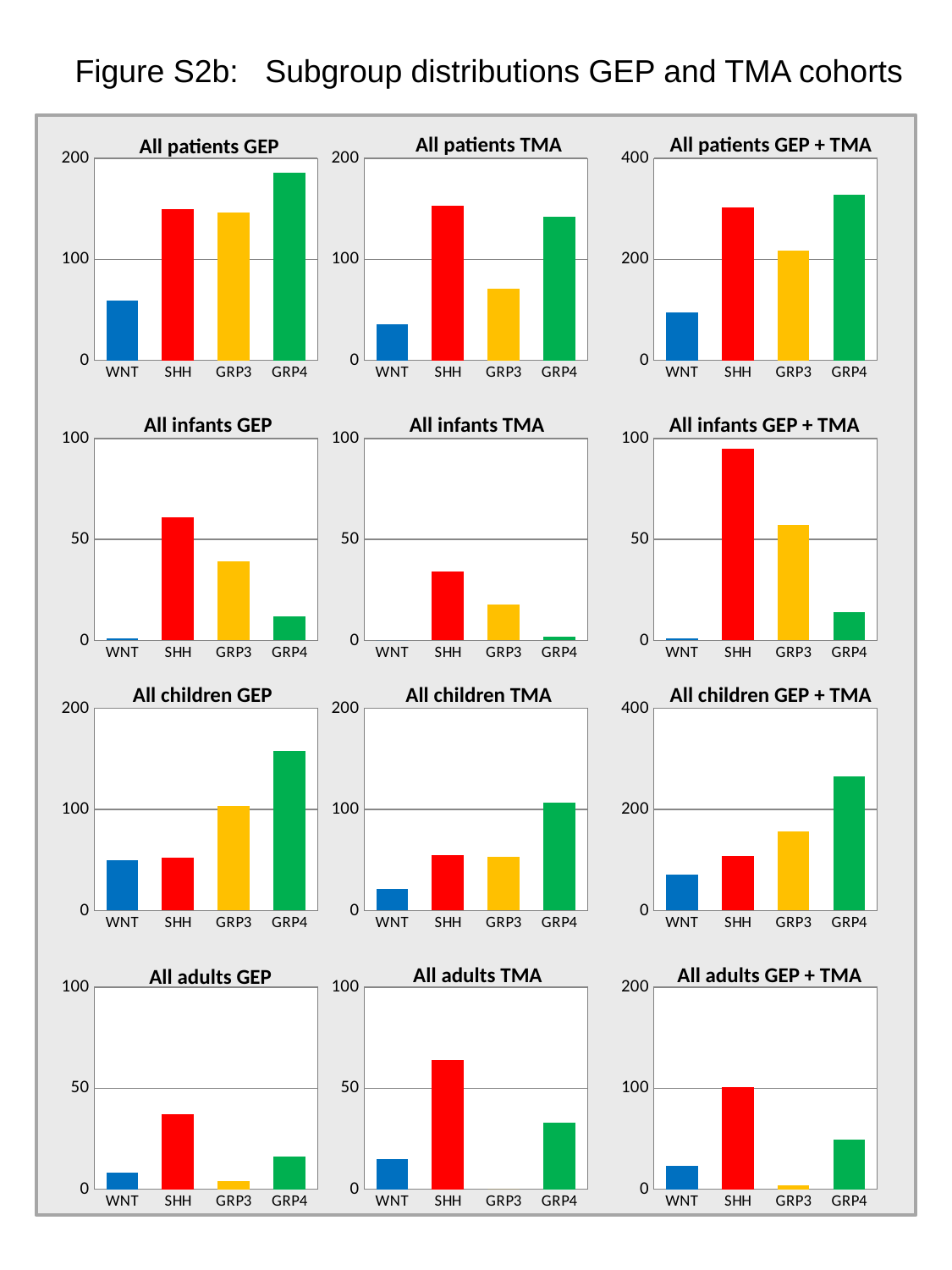

Figure S2b: Subgroup distributions GEP and TMA cohorts
All patients TMA
All patients GEP + TMA
All patients GEP
### Chart
| Category | |
|---|---|
| WNT | 59.0 |
| SHH | 150.0 |
| GRP3 | 146.0 |
| GRP4 | 186.0 |
### Chart
| Category | |
|---|---|
| WNT | 36.0 |
| SHH | 153.0 |
| GRP3 | 71.0 |
| GRP4 | 142.0 |
### Chart
| Category | |
|---|---|
| WNT | 95.0 |
| SHH | 303.0 |
| GRP3 | 217.0 |
| GRP4 | 328.0 |All infants GEP
All infants TMA
All infants GEP + TMA
### Chart
| Category | |
|---|---|
| WNT | 1.0 |
| SHH | 61.0 |
| GRP3 | 39.0 |
| GRP4 | 12.0 |
### Chart
| Category | |
|---|---|
| WNT | 0.0 |
| SHH | 34.0 |
| GRP3 | 18.0 |
| GRP4 | 2.0 |
### Chart
| Category | |
|---|---|
| WNT | 1.0 |
| SHH | 95.0 |
| GRP3 | 57.0 |
| GRP4 | 14.0 |All children GEP
All children TMA
All children GEP + TMA
### Chart
| Category | |
|---|---|
| WNT | 50.0 |
| SHH | 52.0 |
| GRP3 | 103.0 |
| GRP4 | 158.0 |
### Chart
| Category | |
|---|---|
| WNT | 21.0 |
| SHH | 55.0 |
| GRP3 | 53.0 |
| GRP4 | 107.0 |
### Chart
| Category | |
|---|---|
| WNT | 71.0 |
| SHH | 107.0 |
| GRP3 | 156.0 |
| GRP4 | 265.0 |All adults TMA
All adults GEP + TMA
All adults GEP
### Chart
| Category | |
|---|---|
| WNT | 8.0 |
| SHH | 37.0 |
| GRP3 | 4.0 |
| GRP4 | 16.0 |
### Chart
| Category | |
|---|---|
| WNT | 15.0 |
| SHH | 64.0 |
| GRP3 | 0.0 |
| GRP4 | 33.0 |
### Chart
| Category | |
|---|---|
| WNT | 23.0 |
| SHH | 101.0 |
| GRP3 | 4.0 |
| GRP4 | 49.0 |

## Slide 4
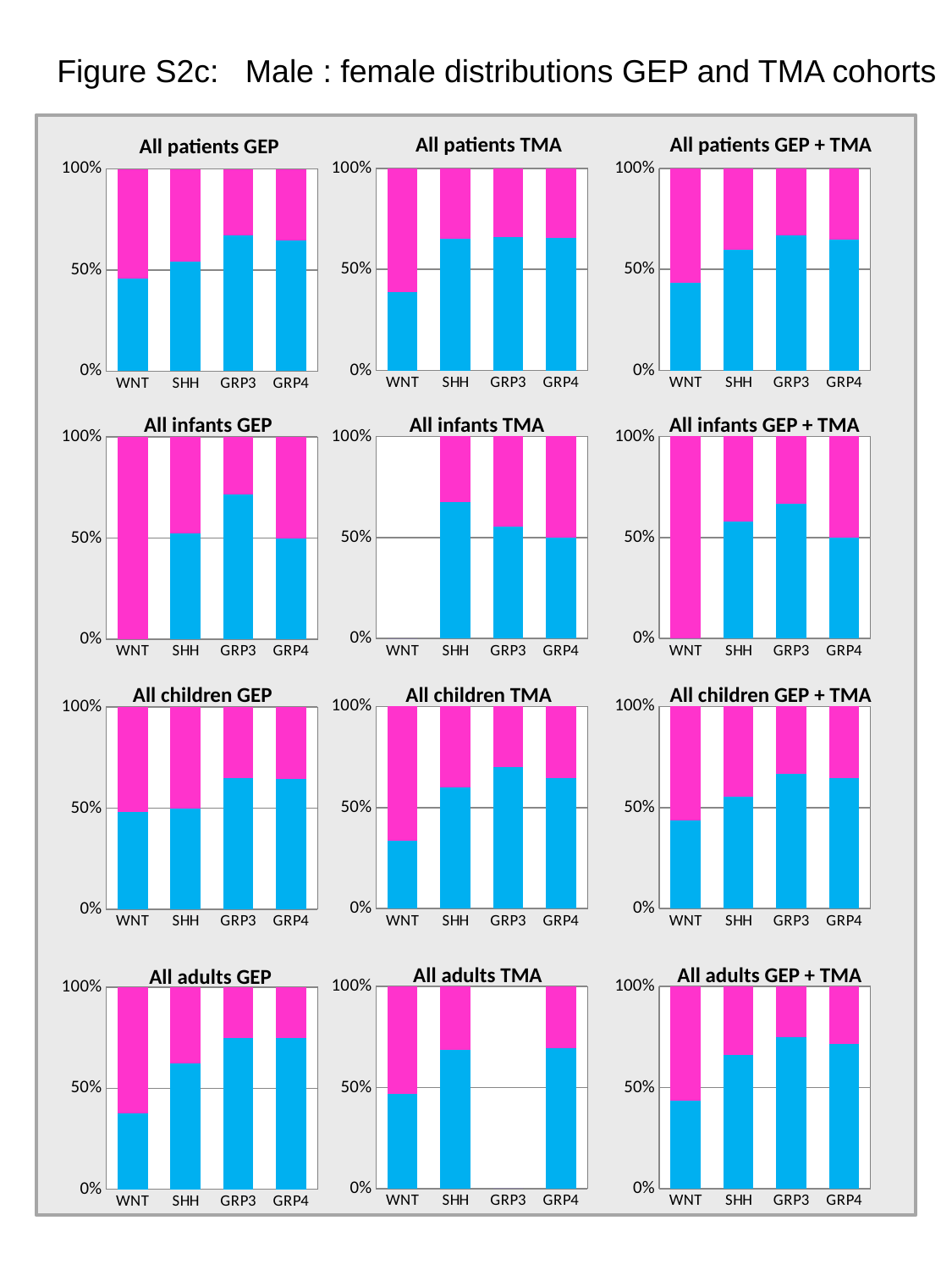

Figure S2c: Male : female distributions GEP and TMA cohorts
All patients TMA
All patients GEP + TMA
All patients GEP
### Chart
| Category | | |
|---|---|---|
| WNT | 14.0 | 22.0 |
| SHH | 100.0 | 53.0 |
| GRP3 | 47.0 | 24.0 |
| GRP4 | 93.0 | 49.0 |
### Chart
| Category | | |
|---|---|---|
| WNT | 41.0 | 54.0 |
| SHH | 181.0 | 122.0 |
| GRP3 | 145.0 | 72.0 |
| GRP4 | 213.0 | 115.0 |
### Chart
| Category | | |
|---|---|---|
| WNT | 27.0 | 32.0 |
| SHH | 81.0 | 69.0 |
| GRP3 | 98.0 | 48.0 |
| GRP4 | 120.0 | 66.0 |All infants GEP
All infants TMA
All infants GEP + TMA
### Chart
| Category | | |
|---|---|---|
| WNT | 0.0 | 0.0 |
| SHH | 23.0 | 11.0 |
| GRP3 | 10.0 | 8.0 |
| GRP4 | 1.0 | 1.0 |
### Chart
| Category | | |
|---|---|---|
| WNT | 0.0 | 1.0 |
| SHH | 55.0 | 40.0 |
| GRP3 | 38.0 | 19.0 |
| GRP4 | 7.0 | 7.0 |
### Chart
| Category | | |
|---|---|---|
| WNT | 0.0 | 1.0 |
| SHH | 32.0 | 29.0 |
| GRP3 | 28.0 | 11.0 |
| GRP4 | 6.0 | 6.0 |All children GEP
All children TMA
All children GEP + TMA
### Chart
| Category | | |
|---|---|---|
| WNT | 7.0 | 14.0 |
| SHH | 33.0 | 22.0 |
| GRP3 | 37.0 | 16.0 |
| GRP4 | 69.0 | 38.0 |
### Chart
| Category | | |
|---|---|---|
| WNT | 31.0 | 40.0 |
| SHH | 59.0 | 48.0 |
| GRP3 | 104.0 | 52.0 |
| GRP4 | 171.0 | 94.0 |
### Chart
| Category | | |
|---|---|---|
| WNT | 24.0 | 26.0 |
| SHH | 26.0 | 26.0 |
| GRP3 | 67.0 | 36.0 |
| GRP4 | 102.0 | 56.0 |All adults TMA
All adults GEP + TMA
All adults GEP
### Chart
| Category | | |
|---|---|---|
| WNT | 7.0 | 8.0 |
| SHH | 44.0 | 20.0 |
| GRP3 | 0.0 | 0.0 |
| GRP4 | 23.0 | 10.0 |
### Chart
| Category | | |
|---|---|---|
| WNT | 10.0 | 13.0 |
| SHH | 67.0 | 34.0 |
| GRP3 | 3.0 | 1.0 |
| GRP4 | 35.0 | 14.0 |
### Chart
| Category | | |
|---|---|---|
| WNT | 3.0 | 5.0 |
| SHH | 23.0 | 14.0 |
| GRP3 | 3.0 | 1.0 |
| GRP4 | 12.0 | 4.0 |

## Slide 5
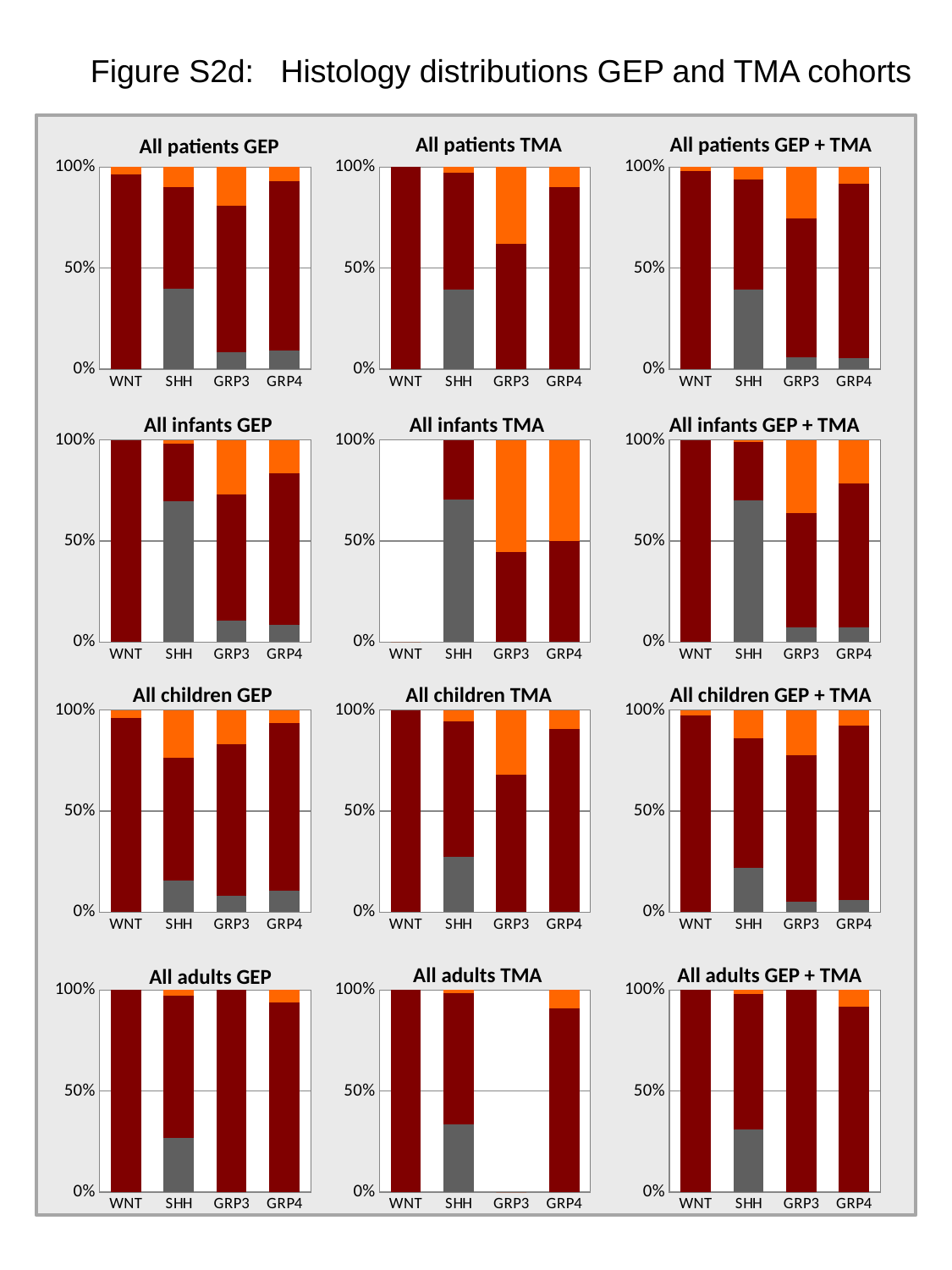

Figure S2d: Histology distributions GEP and TMA cohorts
All patients TMA
All patients GEP + TMA
All patients GEP
### Chart
| Category | | Classic | LCA |
|---|---|---|---|
| WNT | 0.0 | 56.0 | 2.0 |
| SHH | 57.0 | 73.0 | 14.0 |
| GRP3 | 12.0 | 102.0 | 27.0 |
| GRP4 | 17.0 | 152.0 | 13.0 |
### Chart
| Category | | Classic | LCA |
|---|---|---|---|
| WNT | 0.0 | 36.0 | 0.0 |
| SHH | 60.0 | 88.0 | 4.0 |
| GRP3 | 0.0 | 44.0 | 27.0 |
| GRP4 | 0.0 | 128.0 | 14.0 |
### Chart
| Category | | Classic | LCA |
|---|---|---|---|
| WNT | 0.0 | 92.0 | 2.0 |
| SHH | 117.0 | 161.0 | 18.0 |
| GRP3 | 12.0 | 146.0 | 54.0 |
| GRP4 | 17.0 | 280.0 | 27.0 |All infants GEP
All infants TMA
All infants GEP + TMA
### Chart
| Category | | Classic | LCA |
|---|---|---|---|
| WNT | 0.0 | 1.0 | 0.0 |
| SHH | 39.0 | 16.0 | 1.0 |
| GRP3 | 4.0 | 23.0 | 10.0 |
| GRP4 | 1.0 | 9.0 | 2.0 |
### Chart
| Category | | Classic | LCA |
|---|---|---|---|
| WNT | 0.0 | 0.0 | 0.0 |
| SHH | 24.0 | 10.0 | 0.0 |
| GRP3 | 0.0 | 8.0 | 10.0 |
| GRP4 | 0.0 | 1.0 | 1.0 |
### Chart
| Category | | Classic | LCA |
|---|---|---|---|
| WNT | 0.0 | 1.0 | 0.0 |
| SHH | 63.0 | 26.0 | 1.0 |
| GRP3 | 4.0 | 31.0 | 20.0 |
| GRP4 | 1.0 | 10.0 | 3.0 |All children GEP
All children TMA
All children GEP + TMA
### Chart
| Category | | Classic | LCA |
|---|---|---|---|
| WNT | 0.0 | 48.0 | 2.0 |
| SHH | 8.0 | 31.0 | 12.0 |
| GRP3 | 8.0 | 75.0 | 17.0 |
| GRP4 | 16.0 | 128.0 | 10.0 |
### Chart
| Category | | Classic | LCA |
|---|---|---|---|
| WNT | 0.0 | 21.0 | 0.0 |
| SHH | 15.0 | 37.0 | 3.0 |
| GRP3 | 0.0 | 36.0 | 17.0 |
| GRP4 | 0.0 | 97.0 | 10.0 |
### Chart
| Category | | Classic | LCA |
|---|---|---|---|
| WNT | 0.0 | 69.0 | 2.0 |
| SHH | 23.0 | 68.0 | 15.0 |
| GRP3 | 8.0 | 111.0 | 34.0 |
| GRP4 | 16.0 | 225.0 | 20.0 |All adults TMA
All adults GEP + TMA
All adults GEP
### Chart
| Category | | Classic | LCA |
|---|---|---|---|
| WNT | 0.0 | 7.0 | 0.0 |
| SHH | 10.0 | 26.0 | 1.0 |
| GRP3 | 0.0 | 4.0 | 0.0 |
| GRP4 | 0.0 | 15.0 | 1.0 |
### Chart
| Category | | Classic | LCA |
|---|---|---|---|
| WNT | 0.0 | 15.0 | 0.0 |
| SHH | 21.0 | 41.0 | 1.0 |
| GRP3 | 0.0 | 0.0 | 0.0 |
| GRP4 | 0.0 | 30.0 | 3.0 |
### Chart
| Category | | Classic | LCA |
|---|---|---|---|
| WNT | 0.0 | 22.0 | 0.0 |
| SHH | 31.0 | 67.0 | 2.0 |
| GRP3 | 0.0 | 4.0 | 0.0 |
| GRP4 | 0.0 | 45.0 | 4.0 |

## Slide 6
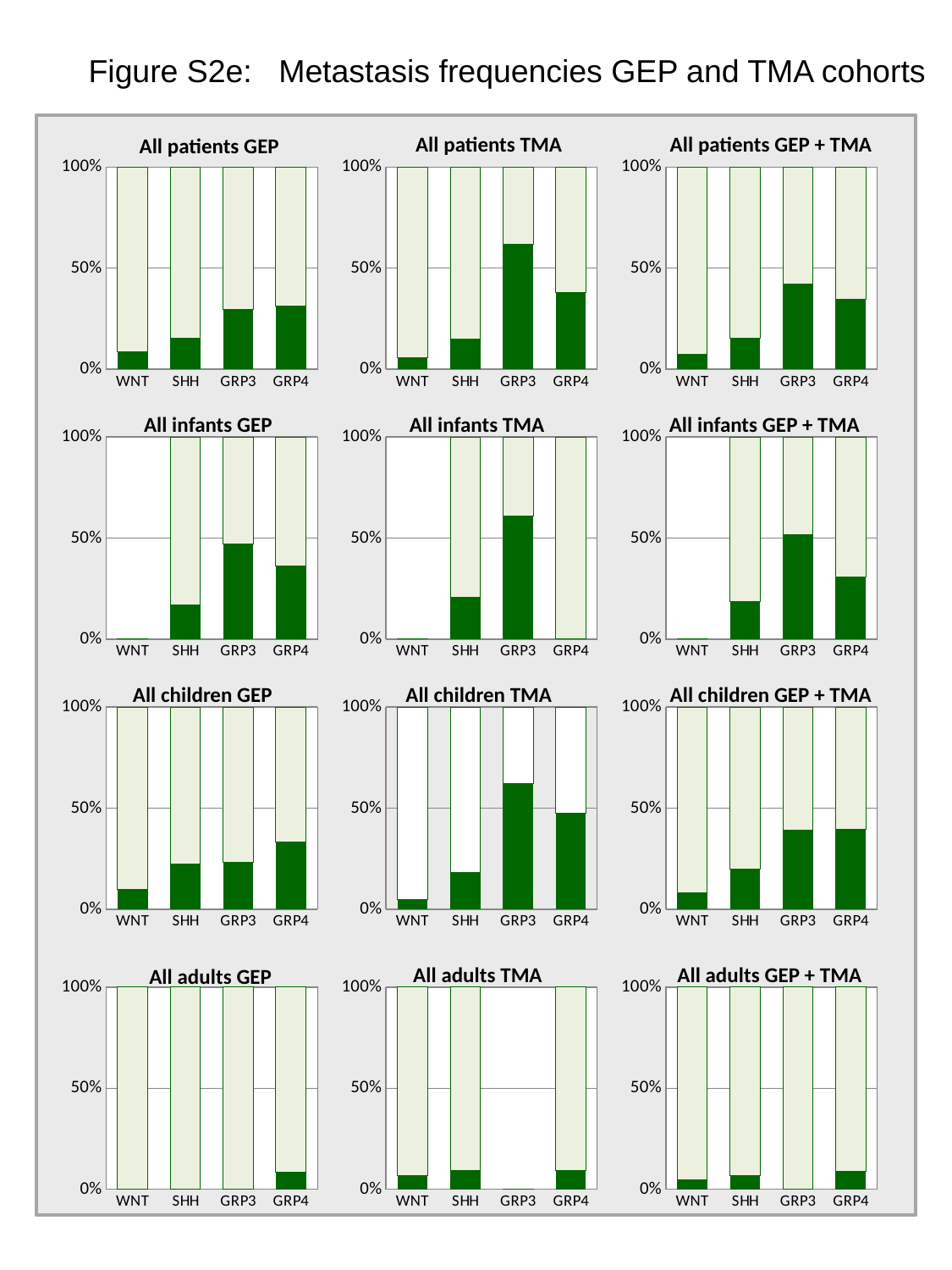

Figure S2e: Metastasis frequencies GEP and TMA cohorts
All patients TMA
All patients GEP + TMA
All patients GEP
### Chart
| Category | M+ | M0 |
|---|---|---|
| WNT | 4.0 | 43.0 |
| SHH | 18.0 | 99.0 |
| GRP3 | 34.0 | 81.0 |
| GRP4 | 47.0 | 103.0 |
### Chart
| Category | M+ | M0 |
|---|---|---|
| WNT | 2.0 | 34.0 |
| SHH | 23.0 | 130.0 |
| GRP3 | 44.0 | 27.0 |
| GRP4 | 54.0 | 88.0 |
### Chart
| Category | M+ | M0 |
|---|---|---|
| WNT | 6.0 | 77.0 |
| SHH | 41.0 | 229.0 |
| GRP3 | 78.0 | 108.0 |
| GRP4 | 101.0 | 191.0 |All infants GEP
All infants TMA
All infants GEP + TMA
### Chart
| Category | M+ | M0 |
|---|---|---|
| WNT | 0.0 | 0.0 |
| SHH | 8.0 | 39.0 |
| GRP3 | 16.0 | 18.0 |
| GRP4 | 4.0 | 7.0 |
### Chart
| Category | M+ | M0 |
|---|---|---|
| WNT | 0.0 | 0.0 |
| SHH | 7.0 | 27.0 |
| GRP3 | 11.0 | 7.0 |
| GRP4 | 0.0 | 2.0 |
### Chart
| Category | M+ | M0 |
|---|---|---|
| WNT | 0.0 | 0.0 |
| SHH | 15.0 | 66.0 |
| GRP3 | 27.0 | 25.0 |
| GRP4 | 4.0 | 9.0 |All children GEP
All children TMA
All children GEP + TMA
### Chart
| Category | M+ | M0 |
|---|---|---|
| WNT | 4.0 | 37.0 |
| SHH | 10.0 | 35.0 |
| GRP3 | 18.0 | 59.0 |
| GRP4 | 42.0 | 85.0 |
### Chart
| Category | M+ | M0 |
|---|---|---|
| WNT | 1.0 | 20.0 |
| SHH | 10.0 | 45.0 |
| GRP3 | 33.0 | 20.0 |
| GRP4 | 51.0 | 56.0 |
### Chart
| Category | M+ | M0 |
|---|---|---|
| WNT | 5.0 | 57.0 |
| SHH | 20.0 | 80.0 |
| GRP3 | 51.0 | 79.0 |
| GRP4 | 93.0 | 141.0 |All adults TMA
All adults GEP + TMA
All adults GEP
### Chart
| Category | M+ | M0 |
|---|---|---|
| WNT | 0.0 | 6.0 |
| SHH | 0.0 | 25.0 |
| GRP3 | 0.0 | 4.0 |
| GRP4 | 1.0 | 11.0 |
### Chart
| Category | M+ | M0 |
|---|---|---|
| WNT | 1.0 | 14.0 |
| SHH | 6.0 | 58.0 |
| GRP3 | 0.0 | 0.0 |
| GRP4 | 3.0 | 30.0 |
### Chart
| Category | M+ | M0 |
|---|---|---|
| WNT | 1.0 | 20.0 |
| SHH | 6.0 | 83.0 |
| GRP3 | 0.0 | 4.0 |
| GRP4 | 4.0 | 41.0 |

## Slide 7
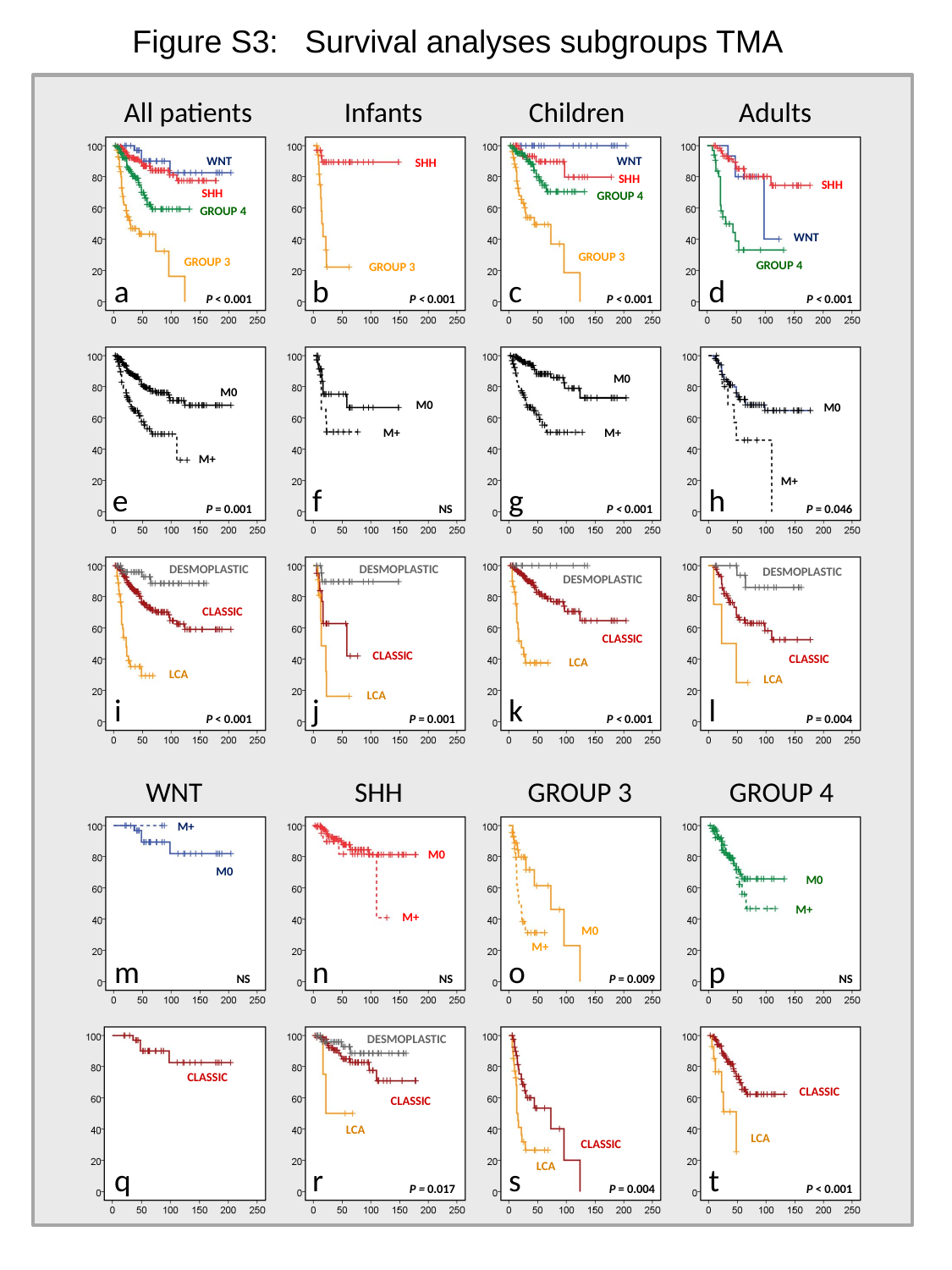

Figure S3: Survival analyses subgroups TMA
All patients
Infants
Children
Adults
WNT
WNT
SHH
SHH
SHH
SHH
GROUP 4
GROUP 4
WNT
GROUP 3
GROUP 3
GROUP 4
GROUP 3
a
b
c
d
P < 0.001
P < 0.001
P < 0.001
P < 0.001
M0
M0
M0
M0
M+
M+
M+
M+
e
f
g
h
P = 0.001
NS
P < 0.001
P = 0.046
DESMOPLASTIC
DESMOPLASTIC
DESMOPLASTIC
DESMOPLASTIC
CLASSIC
CLASSIC
CLASSIC
CLASSIC
LCA
LCA
LCA
LCA
i
j
k
l
P < 0.001
P = 0.001
P < 0.001
P = 0.004
WNT
SHH
GROUP 3
GROUP 4
M+
M0
M0
M0
M+
M+
M0
M+
m
n
o
p
 NS
NS
P = 0.009
NS
DESMOPLASTIC
CLASSIC
CLASSIC
CLASSIC
LCA
LCA
CLASSIC
LCA
q
r
s
t
P = 0.017
P = 0.004
P < 0.001
